# Encapsulation of Fruit Flavor Compounds through Interaction with Polysaccharides

**DOI:** 10.3390/molecules26144207

**Published:** 2021-07-11

**Authors:** Ivana Buljeta, Anita Pichler, Ivana Ivić, Josip Šimunović, Mirela Kopjar

**Affiliations:** 1 Faculty of Food Technology Osijek, Josip Juraj Strossmayer University of Osijek, F. Kuhača 18, 31000 Osijek, Croatia; ivana.buljeta@ptfos.hr (I.B.); anita.pichler@ptfos.hr (A.P.); ivana.ivic@ptfos.hr (I.I.); 2 Department of Food, Bioprocessing and Nutrition Sciences, North Carolina State University, Raleigh, NC 27695, USA; simun@ncsu.edu

**Keywords:** polysaccharides, flavor compounds, encapsulation, interactions

## Abstract

Production and storage, the influence of packaging materials and the presence of other ingredients in fruit products can cause changes in flavor compounds or even their loss. Due to these issues, there is a need to encapsulate flavor compounds, and polysaccharides are often used as efficient carriers. In order to achieve effective encapsulation, satisfactory retention and/or controlled release of flavor compounds, it is necessary to understand the nature of the coated and coating materials. Interactions that occur between these compounds are mostly non-covalent interactions (hydrogen bonds, hydrophobic interactions and van der Waals forces); additionally, the formation of the inclusion complexes of flavor compounds and polysaccharides can also occur. This review provides insight into studies about the encapsulation of flavor compounds, as well as basic characteristics of encapsulation such as the choice of coating material, the effect of various factors on the encapsulation efficiency and an explanation of the nature of binding.

## 1. Introduction

In order for food products to be accepted by consumers, they must, among other requirements, satisfy the organoleptic properties, which are mainly affected by flavor. The flavor compounds must pass from the product to the gas phase via the interface and reach the olfactory epithelium to be perceived during product consumption. The presence of other components (proteins, polysaccharides, lipids) in the product matrix strongly affects the retention and perception of flavor [1].

Flavor compounds are usually organic compounds [2] and include acids, alcohols, ketones, aldehydes, esters, neutral compounds, nitrogen and sulfur compounds and hydrocarbons [3]. These compounds have a low molecular weight (<400 Da), are very sensitive to heat, light and oxygen, have a low boiling point and are highly volatile [4]. Table 1 shows some of the main physicochemical characteristics of flavor compounds that belong to the different chemical classes, as well as fruit sources and flavor descriptors for these compounds.

Most of them have a desirable fragrance and are therefore often used in the food and chemical industries (e.g., beverage, bakery, cosmetics and perfumes industries) [10]. A limiting factor for the application of some of these compounds is their toxicity, and it is of great importance to present some safety information such as LC_50_ (median lethal dose) (Table 2). In addition, they may possess antibacterial, antioxidant and antifungal properties [11].

Production and storage, the influence of packaging materials and the presence of other ingredients in the products cause changes in the flavor compounds or even their loss. Since flavor is an important factor influencing the sale of a particular product and affects consumer satisfaction, there is a need to encapsulate it in order to preserve/partially preserve their native form and prevent the aforementioned problems [12,13,14]. Encapsulation is a technique in which a substance or a mixture of substances is coated or entrapped in another material that forms a protective shell or wall [15,16]. Polysaccharides, as ingredients usually present in foods, can be used as carriers of flavor compounds. They are known for their contribution to the reduction of flavor release because they increase viscosity and/or create molecular interactions with flavor compounds [17] and additionally possess different functional properties and possible health benefits. Starch, as the most important polysaccharide in the food industry, is used as a gelling agent, stabilizer and/or thickener and at the same time can interact with small molecules such as flavor compounds [18]. Dietary fibers are oligosaccharides, polysaccharides and derivatives resistant to digestion and adsorption in the human small intestine, and they can be completely or partially fermented in the large intestine [19,20,21]. They are classified into soluble and insoluble fibers. Soluble dietary fibers are pectins, β-glucans, oligosaccharides, gums, mucilage and inulin. Insoluble dietary fibers include cellulose, hemicellulose, chitin and resistant starch [21,22]. There are many positive health effects attributed to dietary fibers, such as reduced hypertension, hyperlipidemia, obesity, constipation and type II diabetes [23,24,25]. Additionally, foods enriched with dietary fibers had better properties (increased water retention capacity, gel formation, viscosity, fermentability or adsorption) [22].

The effect of polysaccharides on flavor is complex because they can have an effect on both the retention and release of the flavor. They can directly capture flavor compounds and thus retain them while at the same time they cause textural and physicochemical changes in the matrix and so alter the release of the flavor [5]. The rate of release of flavors from the product depends on flavor compounds volatility, which is a thermodynamic factor, but also on the resistance to mass transfer from the product to the air, which is a kinetic factor [26].

Increased retention of flavor compounds (from the same group) by polysaccharides was observed at higher molecular weight while increased volatility and polarity decreased retention. It is interesting to mention that the crystallization of the carrier led to reduced retention of the flavor compounds because of a cross-linking effect that was created and caused a reduction in the surface area between the polymer chains. In this way, flavor compounds were forcibly squeezed out from the matrix to the surface [27].

Non-aromatic compounds in foods (such as polysaccharides) can have a major impact on the overall flavor since they can affect the rate and extent of flavor release. The binding of flavor compounds can lead to an imbalance of the flavor profile, and it is necessary to know the mechanism of release of flavor components from the product matrices, especially when new products are developed. Additionally, it is important to know the nature of the interactions that occur between polysaccharides and flavor compounds, and this review will provide a better insight into this topic.

## 2. Encapsulation and Selection of Flavor Compounds Carriers

### 2.1. Encapsulation

Encapsulation is a technology developed in the 1960s, and the purpose of encapsulation was the retention of the flavor compounds during storage of products, protection from oxidation reactions, the extension of the shelf-life of the flavor and controlled release [28,29,30]. The encapsulated material (pure substances or a mixture) is known as coated material, core material, payload, internal phase, fill or actives, while the coating material is called a shell, wall material, capsule, carrier, membrane, film, the outer shell or packing material [16,31]. Different shapes of microcapsules can be formed, and their morphology depends on the arrangement of the coated material and the deposition process of the wall material. In the food and pharmaceutical industries, microencapsulation has been widely used, while recently nanoencapsulation had attracted increasing attention due to better encapsulation efficiency, stability and more successful controlled release of the encapsulated material [30]. The obtained encapsulates can be in the form of powder, liquid or paste, depending on the applied technique. Their purpose is different; for example, the powder form of encapsulated flavor is mainly used in the bakery industry [30].

Different methods for the encapsulation of food ingredients are available, and they include: emulsification (high-pressure homogenization, microfluidization, ultrasonic technique, spontaneous emulsification, phase inversion emulsification, miscellaneous emulsification techniques), spray drying, spray chilling/cooling, electro-spinning and electro-spraying, freeze-drying, spray-freeze-drying, extrusion, coacervation, fluid bed coating and molecular inclusion in cyclodextrins. They can be divided into chemical (e.g., molecular inclusion), physico-chemical (e.g., emulsification) and physico-mechanical methods (e.g., freeze-drying) [30].

### 2.2. Flavor Carriers

The selection of a coating material depends on its nature, application of the final product and the encapsulation process [30]. The most common carriers of flavor compounds are polysaccharides (e.g., maltodextrins, β-cyclodextrins, modified starches), proteins, lipids, synthetic polymers and their combinations [32,33]. The process of selecting flavor compounds carriers is demanding and should be carried out carefully in order to avoid negative consequences. In addition to this, some flavor compounds are poorly retained while some are firmly entrapped, resulting in an uneven flavor. This is a challenge for the food industry, and solutions can be found in the understanding of interactions that take place between the flavor compounds and their carriers [32]. The importance of the carrier selection for the encapsulation efficiency of certain flavor compounds was shown in a study by Zhang et al. [4] where ethyl butyrate and hexanal were encapsulated by β-cyclodextrin and γ-cyclodextrin. It was observed that the ethyl butyrate/γ-cyclodextrin complex had the highest inclusion efficiency followed by ethyl butyrate/β-cyclodextrin, hexanal/β-cyclodextrin and hexanal/γ-cyclodextrin complexes. There are many factors that affect the formation of complexes, such as matching cyclodextrin cavity and payload molecules and the polarity of payload molecules, environmental factors. In this case, for ethyl butyrate, the cavity of γ-cyclodextrin was more suitable, while for hexanal, β-cyclodextrin was better. Ethyl butyrate is a less polar molecule than hexanal, and the aqueous complexation medium that was used favored the entry of less polar payload molecules into the hydrophobic cavity of cyclodextrin. Further, it was observed that during storage, hexanal complexes had better stability [4]. It can be observed that each component will behave differently during encapsulation but also during storage, depending on the carrier material. Additionally, a contribution that can be achieved through the incorporation of flavor compounds into a food product is of high importance for the quality of the particular product, but also for consumer satisfaction in general [33].

### 2.3. Flavor Retention

Retention of flavor during the encapsulation process depends on the following factors [30,34,35,36,37,38,39,40,41]:

- Flavor compound characteristics: the type of compounds (e.g., ketones, esters, acids), polarity, size, molecular weight, relative volatility, concentration;
- Carrier characteristics: the type of carrier (e.g., proteins, fats, polysaccharides), molecular weight, viscosity, solids content, glass transition temperature, solubility, emulsifying ability, film-forming capability, concentration, biocompatibility;
- The method of sample preparation: emulsification method, emulsion droplet size, emulsion viscosity, emulsion stability;
- Applied encapsulation methods (e.g., spray-drying, extrusion) and
- Operating parameters during encapsulation: feed flow rate, inlet and outlet air temperature, air flow rate, type of atomizer.

In the food industry, polysaccharides are used as sweeteners, thickeners and/or gelling agents. Due to their broad application range, they are often found in foods together with flavor compounds and have a strong impact on them. There are many properties of flavor compounds that affect their interactions with other components present in foods, and those that need to be emphasized are the molecular size, shape, volatility and functional groups [3].

## 3. Polysaccharides–Flavor Interactions

### 3.1. Starch–Flavor Compounds Interactions

Starch is one of the main food components of plant origin used as an additive for thickening and stabilization. It is composed of glucopyranose units, and its main components are amylose (linear polymer) and amylopectin (short, branched chains), whose ratio affects the physical properties of a particular starch. Furthermore, starches can also serve as carriers for encapsulation of flavor compounds and thus contribute to flavor quality [42,43]. Starch interacts with flavor compounds, and these interactions depend on various factors such as polarity, molecular weight, hydrophobicity, volatility and solubility of flavor compounds, nature of the starch and competition among flavor compounds [43,44,45]. The formation of helical inclusion complexes was reported as an example of the specific binding of starch (especially the linear amylose) and flavor compounds in such a way that the flavor molecules are wrapped in a left-handed single helical structure [10,46]. These types of complexes with starch were reported for aldehydes, alcohols, terpenes, ketones and fatty acids [42,47]. Although it was stated that amylopectin might also be partially involved in complex formation, other studies indicated that starch with a higher amylopectin content did not form inclusion complexes or form a less [48,49]. The second type of interaction between starch and flavor compounds included polar interactions. It was emphasized that hydrogen bonds were formed between the hydroxyl groups of starch and flavor compounds [16,50,51]. Additionally, powdered starch is porous on its surface, and the flavor compounds can be retained on such surfaces by physical sorption [50]. This mode of interaction is more important for high concentrations of flavor compounds [46]. Sometimes flavor retention cannot be explained by the formation of an inclusion complex with amylose, and in those cases, interactions with amylopectin due to adsorption (including hydrogen bonds) are more likely to occur [5].

In the study conducted by Tietz et al. [46], there were no competitive interactions of flavor compounds and starch, and a possible reason for this was that very low concentrations of flavor compounds had been used, while the binding capacity of starch is 100 times higher. Furthermore, in systems with low water content and different types of starch, retention of flavor increased with the polarity of flavor compounds. Additionally, granular starch retained less flavor, and the reason may be in the lower availability of starch molecules [27]. Table 3 provides an overview of studies that investigated the formation of complexes between flavor compounds and starch and their interactions.

### 3.2. Maltodextrin–Flavor Compounds Interactions

Maltodextrin is obtained by acid or enzymatic hydrolysis of starch from various sources (corn, potato or others) and is a relatively inexpensive polysaccharide with a neutral taste [60]. It has low viscosity, high water solubility and is often used as an encapsulating material for the purpose of protecting flavor compounds from physical and chemical changes [61]. The main disadvantage in its application is its weak emulsifying capacity and low retention of flavor compounds [28], so it is often used in a mixture with other polysaccharides. Commercially, maltodextrin is available in different dextrose equivalent (DE) grades, which represent the degree of starch hydrolysis. Previous studies had shown that flavor retention depended on the DE of maltodextrin, but later it was found that use of the DE value was not adequate for predictions of maltodextrins performance [16]. Additionally, it has been observed that temperature, as one of the environmental factors, may have an effect on the retention or release of flavor compounds in polysaccharide systems. In maltodextrin solution (DE 5; 10%, *w*/*w*), retention of studied compounds (1-hexanol, 2-butanone, 2-hexanone, hexanal, 2-octanone, ethyl butanoate, 2-heptanone) was better at higher temperatures (80 °C), indicating a hydrophobic effect [62]. As for the mechanisms responsible for the interactions of maltodextrin and flavor compounds, mechanisms described for starch, involving the formation of an inclusion complex and polar interactions, can also be applied in this case [3,63]. Table 4 shows some studies that examined the effect of maltodextrin on flavor compounds.

### 3.3. Pectin-Flavor Compounds Interactions

Pectin is a complex mixture of polysaccharides, consisting of D-galacturonic acid units linked by α (1→4) glycosidic bonds [71]. It is present in the middle lamella of the cell wall of fruits and vegetables. Monomeric sugar molecules (galactose, arabinose or rhamnose) that are esterified or acetylated are responsible for the heterogeneous structure of pectin [72]. The use of pectin is as an agent for gelling, thickening, stabilizing, emulsifying purposes, and can be applied in both food and pharmaceutical industries [73,74,75]. According to the degree of esterification (DE), pectins are divided into high-esterified (DE > 50%) and low-esterified (DE < 50%) pectins [75]. Due to its carboxyl groups, pectin is an ionic molecule, and therefore, its thickening properties depend on the degree of esterification, pH, the presence of bivalent metal ions and the arrangement of free carboxyl groups [76].

In a study conducted by Guichard et al. [77], it was observed that the addition of pectin to jam had the effect of reducing the intensity of taste, which can be explained by the slower diffusion of flavor compounds trapped in the pectin. To describe the interactions between flavor compounds and pectin, Braudo et al. [78] investigated four commonly present flavor compounds in foods (2-acetyl pyridine, 2-acetyl tiophene, 2,3-diethyl pyrazine and 2-octanone) and low-esterified pectin. Heterocyclic flavor compounds were not adsorbed on low-esterified pectin in a neutral medium, but in an acidic medium; the adsorption took place through hydrogen bonds. These bonds were formed between the aromatic ring of the flavor compound and the hydrogen atoms on the carboxyl group of pectin. In the neutral medium, 2-octanone was adsorbed on low-esterified pectin by van der Waals interactions, while in the acidic medium, the adsorption took place via hydrophobic interactions. It was observed that the binding of heterocyclic flavor compounds to low-esterified pectin increased with decreasing pH (from 4.0 to 3.0), while at pH 3.0, the maximum was reached at a concentration of 0.6–0.7% of low-esterified pectin, followed by a decrease. Two explanations for this phenomenon were proposed: binding of pectin molecules to each other and binding of flavor compounds to pectin, and both of them included hydrogen bonds. Additionally, this group of scientists examined the effect of essential metal ions (Ca^2+^, Zn^2+^, Mg^2+^ and Fe^3+^) on the nature of binding of previously mentioned flavor compounds to low-esterified pectin. It was observed that metal ions prevented the adsorption of heterocyclic flavor compounds on low-esterified pectin in an acidic medium due to their interaction with carboxyl groups of low-esterified pectin. The carboxyl groups of pectin that interacted with metal ions were not able to form hydrogen bonds with flavor compounds. The presence of metal ions inhibited the binding of 2-octanone to low-esterified pectin in acidic media, while in neutral media, it had no effect [78].

There is an increasing demand for low-fat products in the marketplace, but there are changes in flavor release, flavor perception, structure and appearance in such products. To make up for the shortcomings caused by fat removal, fat substitutes such as maltodextrin, starch and carrageenan are used. In a study conducted by Tromelin et al. [1], the effect of pectin and carrageenan on flavor retention was examined. It was determined that *iota*-carrageenan altered the interactions of water molecules and flavor compounds (ethyl propanoate, ethyl trans-2-butenoate, 3-methylbutyl acetate, isopropyl 2-methyl-2-butenoate, ethyl hexanoate, ethyl heptanoate, butyl pentanoate, 4-methylpent-3-en-2-one, 4-methylpentan-2-one, heptan-2-one, 2,6-dimethylheptan-4-one, trans-2-methyl-2-butenal, 2-ethylbutanal), while pectin caused weak changes in these interactions.

### 3.4. Cyclodextrin-Flavor Compounds Interactions

Cyclodextrins are naturally occurring cyclic oligosaccharides composed of α-D-glucopyranose units linked by α (1→4) glycosidic bonds. α-(6 α-D-glucopyranose units), β-(7 units) and γ-cyclodextrins (8 units) are the most important for the food industry [79]. For better illustration, it can be said that cyclodextrin is a “shallow truncated cone”, whose cavity can serve to form complexes with other molecules. The outer surface of cyclodextrins is hydrophilic while their interior, i.e., the cavity, is hydrophobic [80]. Due to its specific structure, cyclodextrin has the ability to form non-covalent bonds with various organic and inorganic (guest) molecules [14]. An interesting fact is that the depth of the cavity in cyclodextrins is approximately equal while the diameter of the cavities increases in α-cyclodextrin, β-cyclodextrin and γ-cyclodextrin, respectively [14,81]. An example of the effect of a larger cavity diameter in β-cyclodextrin than in α-cyclodextrin was shown in the measurement of the binding constants of 14 flavor compounds on these two cyclodextrins. The binding constant (determined by UV/Vis spectroscopy) of all flavor compounds (maltol, furaneol, methyl cinnamate, vanillin, cineole, geraniol, citral, camphor, menthol, eugenol, nootkatone, limonene, p-vinil and guaiacol) to β-cyclodextrin was higher than was the case of α-cyclodextrin. This phenomenon was explained by hydrophobic/hydrophilic interactions [14].

β-cyclodextrins have the ability to protect flavor compounds from oxidation and also from chemical and thermal degradation. In a study by Goubet et al. [32], six flavor compounds (benzyl alcohol, 2-methylbutyric acid, hexanoic acid, hexanol, ethyl hexanoate, ethyl propionate) and their retention on β-cyclodextrin were studied. They observed that the retention of benzyl alcohol was 2 mol/mol of β-cyclodextrin, while for the other five aliphatic flavor compounds, the retention was 1 mol/mol of β-cyclodextrin, which would mean that the aromatic ring had an effect on binding with β-cyclodextrin. Similar results were obtained in the research of Sanemasa et al. [82]. They observed that the retention of benzene and fluorobenzene was 1.9 mol/mol of β-cyclodextrin, while the retention of heptane and pentane was approximately 1 mol/mol of β-cyclodextrin (more precisely, 0.88 and 1.1 mol/mol of β-cyclodextrin, respectively) [82].

Tobitsuka et al. [83] conducted a study to obtain data on the retention of pear [La France (*Pyrus communis* L.)] flavor compounds in cyclodextrin, i.e., to investigate the interactions between aliphatic acetate esters and cyclodextrin. Structural analysis of complexes (α-cyclodextrin-butyl acetate and α-cyclodextrin-hexyl acetate) by NMR spectroscopy showed that there were cross-peaks between protons in α-D-glucopyranose and protons of the alkyl chain of esters. The aforementioned α-D-glucopyranose protons are located within the cyclodextrin cavity (at the 3- and 5-position), indicating that the esters were included in the α-cyclodextrin cavity.

Sometimes it is necessary to remove some compounds from the final product because they can cause undesirable off-flavors. Such problems can occur during the preservation of watermelon juice by thermal treatments. Yang et al. [84] tried to remove unwanted flavor compounds of watermelon juice using β-cyclodextrin, xanthan gum, sugar/acid and carboxymethyl cellulose sodium. It has been shown that β-cyclodextrin had the best effect on improving the sensory quality of watermelon juice by effectively reducing 1-octanol, (*E*)-2-octanol, decanal and (*E*)-2-decenal by 22.81%, 36.43%, 50.82% and 28.19%, respectively. Changes in intensity of stretching vibration bands determined by FT-IR analysis suggested the formation of non-covalent bonds (hydrogen bonds) between β-cyclodextrin and (*E*)-2-decenal or 1-octanol.

Cyclodextrins are some of the most common carriers of flavor compounds, and Table 5 presents an overview of flavor compounds complexed with different types of cyclodextrins.

### 3.5. Guar Gum-Flavor Compounds Interactions

Gums are used to increase product viscosity, and it has been observed that highly volatile components were greatly affected by changes in viscosity [76,100]. The addition of carboxymethyl cellulose or guar gum reduced the volatility of highly volatile non-polar flavor compounds such as α-pinene, 1,8-cineole, ethyl 2-methylbutyrate, while less volatile components such as 2-methoxy-3-methylpyrazine, vanillin, methyl anthranilate and maltol did not exhibit this phenomenon [76]. Additionally, the addition of guar gum or carboxymethyl cellulose reduced the release of limonene, hexanal, dimethyl sulfide, ethylbenzene, ethylsulfide, hexanone and styrene from model flavor solutions [100]. A group of scientists studied the effect of guar gum on the retention of flavor compounds. Guar gum samples differed in the galactose/mannose ratio, which can be attributed to different origins of plant seeds from which the guar gum was extracted. It was observed that the retention of ethyl hexanoate significantly depended on the galactose/mannose ratio, which can be attributed to the influence of hydrogen bonds. The retention of ethyl decanoate (which is a non-polar molecule) depended on the molecular weight of guar gum and hydrophobic interactions [101].

### 3.6. Gum Arabic–Flavor Compounds Interactions

Gum arabic is one of the common materials used to encapsulate flavor compounds due to its solubility, good retention of flavor compounds and low viscosity. It is also important to note that it is more expensive than some other materials (such as maltodextrin), which limits its use in the food industry [102,103,104]. Interactions between flavor compounds and gums take place via hydrogen bonds, and their applications in food products are for flavor stabilization [3]. During the encapsulation of a mixture of ethyl butyrate, orange oil, ethyl propionate, benzaldehyde and cinnamic aldehyde in maltodextrin and gum arabic, better retention occurred when the proportion of gum arabic increased [28]. In a study conducted by Apintanapong and Noomhorm [105], 2-acetyl-1-pyrroline was encapsulated by spray drying into maltodextrin and gum arabic, which served as a coating material, and the highest quality of the obtained capsules was achieved at a ratio of 30:70 of maltodextrin:gum arabic. Interactions that took place between guar gum and flavor compounds have also been studied by other scientists [106,107,108], and they concluded that the creation of hydrophobic interactions within complexes was responsible for flavor retention.

### 3.7. Xanthan–Flavor Compounds Interactions

Xanthan gum is an extracellular biopolymer made up of D-glucose units linked by β-1,4 bonds as the main chain and two molecules of mannose and one molecule of glucuronic acid as linear side branches. It is able to form an internal hydrophobic region into which flavor compounds can fit and thus prevent their release [109]. As the possible bond between xanthan and 1-octen-3-ol, the formation of hydrogen bond between the hydroxyl group of 1-octen-3-ol and oxygen from carbonyl on xanthan can occur [110]. Guichard and Etiévant [111] made the same assumption for the binding of xanthan and 1-octen-3-ol, and also assumed the formation of hydrogen bonds between the hydroxyl groups on xanthan and oxygen on 2-acetyl pyrazine. In a study conducted by Yang et al. [109], the effect of xanthan on the release of flavor compounds was examined. The results obtained by SPME/GC-MS analysis indicated that the xanthan solution inhibited the release of flavor compounds, but differences between the individual components were also observed due to their different hydrophobicity (different logP value). Terpenes (D-limonene and α-pinene) were retained highly in xanthan solutions, while aldehydes (hexenal and perillaldehyde) and esters (ethyl acetate and ethyl butyrate) are smaller hydrophobic molecules that have been released better from hydrophilic xanthan solutions. In a study by Kopjar et al. [112], retention of eugenol and linalool in hydrogels prepared with hydrocolloids (guar gum and xanthan) and the addition of trehalose and sucrose were investigated. It was observed that samples containing xanthan had lower retention of tested flavor compounds compared to the samples with guar gum. One reason for that may be the trapping of flavor compounds in the hydrophobic cavity of xanthan [113], but also during the preparation of hydrogels, hydrocolloids, sugars and water form complexes may exhibit different affinities for flavor compounds. Studies have shown that ethyl butanoate [101] and methyl butanoate [113] did not interact with xanthan, probably due to their low molecular weight and high solubility. The retention of ethyl hexanoate and ethyl decanoate in xanthan solutions was influenced by the proteins presented in xanthan, while molecular weight showed no effect. Additionally, the content of xanthan acetate groups had an effect on ethyl hexanoate [101].

Another study was conducted on the effect of adding xanthan gum to cloudy apple juice. Untrained panelists observed a difference even with a low amount (0.5 g/L) of added xanthan gum and negatively rated the sample. They observed that with the addition of xanthan gum, the originality of apple juice and its typical flavor were lost [114].

### 3.8. Cellulose-Flavor Compounds Interactions

D-glucose units associated with β (1→4) linkages are the building blocks of cellulose. As the most abundant polysaccharide, cellulose is often used in various industries (chemical, food, biological and others). Due to its neutral taste and desirable effects on digestion, it is suitable as a food additive [115]. Unfortunately, cellulose application in the flavor industry is limited due to the strong intermolecular and intramolecular hydrogen bonds as well as van der Waals forces. Although flavor compounds can be adsorbed into the voids of cellulose chains, retention of these compounds is limited because of the reduced adsorptive capability of cellulose [115]. For these reasons, cellulose derivatives such as hydroxyethyl cellulose, carboxymethyl cellulose [100] and hydroxypropyl methylcellulose [116] were used for flavor compounds encapsulation.

Regenerated porous cellulose particles (RPC) increased the adsorption capacity of cellulose, and the possible use of such materials for encapsulation of L-menthol was investigated. Carboxymethyl cellulose (CMC) was used as the coating layer for RPC, and analyses (FTIR, SEM, G-C) showed that CMC had no effect on menthol content and encapsulation efficiency, while a positive effect on menthol retention during storage was observed. Finally, applied modifications indicated the possible use of these materials for encapsulation [115].

Vukoja et al. [33] carried out the complexation of raspberry juice flavor compounds with cellulose. It turned out that cellulose was a good carrier for flavor compounds of raspberry juice. Interactions that occurred between cellulose and flavor compounds might be through hydrogen bonds and van der Waals forces. The formation of hydrogen bonds was enabled because of hydrophilic regions of cellulose, which are formed when cellulose hydroxyl groups bind to the glucopyranose ring. Van der Waals forces are the result of hydrophobic regions formed due to the binding of –CH groups to the glucopyranose ring [33,117].

### 3.9. β-Glucan–Flavor Compounds Interactions

β-glucans are a group of polysaccharides composed of D-glucopyranosyl units connected by β (1→3) and β (1→4) linkages. They can be found in the cell walls of barley, oats, rye and wheat, but also in fungi, yeasts, algae and bacteria [118]. At neutral pH, they are soluble in water and form a viscous solution [72]. In a study conducted by Christensen et al. [119], the release of 12 esters and 3 alcohols selected from strawberry flavor in an aqueous solution with oat and barley β-glucan (5, 10 and 15%) was examined. The increased concentration of β-glucan and higher molecular weights of the flavor components increased the retention of alcohols and esters. Better retention increase was observed with an increase in β-glucan concentration from 5% to 10% than in the range from 10% to 15%, and oat β-glucan showed better retention of flavor compounds compared to barley β-glucan. Additionally, lower alcohol retention was attributed to the fact that alcohols can be hydrogen acceptors and donors while esters are only hydrogen donors [119].

### 3.10. Glucomannan–Flavor Compounds Interactions

Konjac glucomannan is a linear polysaccharide consisting of mannose units linked by β (1→4) bonds and glucosyl residues which are acetylated 5 to 10%. It has the ability to form highly viscous solutions at low concentrations, and the presence of acetyl groups in the chain inhibits the formation of intramolecular hydrogen bonds, which improves its solubility [120]. Further, it creates interactions synergistically with starch, carrageenan, xanthan and gellan gum, and due to its high water holding capacity, it prevents syneresis in starch gels and reduces the retrogradation degree of starch [18]. The effect of konjac glucomannan (combined with and without potato starch) on flavor compounds was examined in a study by Lafarge et al. [18] using three flavor compounds (ethyl acetate, ethyl hexanoate and carvacrol). In the retention of ethyl hexanoate, there was no difference between the three different dispersions used (potato starch, konjac glucomannan and potato starch–konjac glucomannan combination), which indicated that there was no specific interaction of this component with the tested polysaccharides. Ethyl acetate had over 50% lower retention in the konjac glucomannan dispersion (compared to the other two), and a possible reason for this was the lower concentration of polysaccharides in that dispersion. The addition of konjac glucomannan to the potato starch dispersion reduced carvacrol retention, i.e., reduced the amylose–flavor interaction due to the inhibition of starch granule swelling.

The retention of flavor compounds from different chemical groups onto polysaccharides is different for each group. In a study by Bylaite et al. [121], it was observed that the addition of λ-carrageenan had an effect on the release of flavor compounds from the λ-carrageenan-thickened solution. Although the release rate decreased for all flavor compounds, differences were observed between different chemical groups so that esters had the highest decrease in the release rate, followed by aldehydes, ketones and alcohols, respectively. The explanation for the most pronounced decreased release rate of esters could be that decrease in flavor compounds diffusion rate occurred through macromolecule entanglement due to weak interactions between the λ-carrageenan chains and esters. In contrast, other studies have shown that alcohols were usually best retained onto polysaccharides due to the formation of glycosidic bonds. The sorption of propanol and 1-hexanol onto β-cyclodextrin was higher than the sorption of ethyl acetate and diacetyl [122]. Further, alcohols retention was best in high amylose starch following by acids and aldehydes [3].

## 4. Conclusions

Polysaccharides and flavor are components present in food products, and their interactions may affect product quality. Flavor compounds are highly valuable bioactive substances, which need to be preserved from losses. It is therefore necessary to enable their controlled release in food products. For this purpose, encapsulation of flavor compounds is applied, the efficiency of which is influenced by various factors. When applying one of the encapsulation techniques, it is essential, among other things, to have a detailed understanding of the nature of the coating and coated materials. Interactions that take place during this process, but also subsequently, will affect the stability, i.e., retention and release of the trapped compound. According to published studies, these interactions take place mainly through non-covalent interactions, but also an inclusion complex can be formed. Although numerous studies have been conducted on this topic, understanding and clarification of these interactions continue to be a work in progress, with a need for further research and improvement.

## Figures and Tables

**Table 1:** Physicochemical parameters, fruit source and descriptors of flavor compounds (adapted from [5,6,7,8,9]).

Flavor Compounds	Formula	MW (g/mol)	logP (o/w)	VP	Fruit Source	Flavor Descriptors
Acids						
Acetic acid	C_2_H_4_O_2_	60.05	−0.170	15.700	Grape, blueberry, pineapple	Vinegar, sour
Butanoic acid	C_4_H_8_O_2_	88.11	0.790	1.650	Strawberry, fruit of the deciduous palm Dalieb	Rancid, aged cheesy, butter
2-Methylbutanoic acid	C_5_H_10_O_2_	102.13	1.180	0.554	Bilberry, blueberry, cranberry, wild strawberry	Fruity, cheesy
3-Methylbutanoic acid	C_5_H_10_O_2_	102.13	1.160	0.554	Apple, banana, blackberry, sour cherry, citrus fruits, currant, grape, papaya, peach, pear, red raspberry	Cheesy
*trans*-2-Hexenoic acid	C_6_H_10_O_2_	114.14	1.677	0.054	Apple, banana, red raspberry, wild strawberry	Fatty, rancid
Hexanoic acid	C_6_H_12_O_2_	116.16	1.920	0.158	Overripe guava fruits, strawberry, noni fruit	Rancid, sour, cheesy
Octanoic acid	C_8_H_16_O_2_	144.21	3.050	0.022	Raw earth-almond, noni fruit	Sour, cheesy, rancid, fatty
Decanoic acid	C_10_H_20_O_2_	172.27	4.090	15.000	Apple, banana, cherry, grape, orange, papaya, peach, pear	Rancid, soapy
Alcohols						
Ethanol	C_6_H_6_O	46.07	−0.190	44.600	Orange, mandarin, tangerine	Alcoholic
1-Propanol	C_3_H_8_O	60.10	0.250	26.317	Apple, babaco fruit	Alcoholic
2-Butanol	C_4_H_10_O	74.12	0.610	18.300	Apple, pear	Alcoholic
2-Methyl-1-propanol	C_4_H_10_O	74.12	0.760	9.000	Apple, currant, apricot, banana, sweet cherry	Alcoholic, nail polish
1-Butanol	C_4_H_10_O	74.12	0.880	6.700	Apple, mulberry	Alcoholic
2-Methyl-3-buten-2-ol	C_5_H_10_O	86.13	0.892	22.939	Bilberry, cherimoya, cranberry, black currant, mango	Herbaceous
1-Penten-3-ol	C_5_H_10_O	86.13	0.991	11.179	Banana, blueberry, black currant	Green
3-Methyl-3-buten-1-ol	C_5_H_10_O	86.13	1.098	10.245	Barbados cherry, apple, blackberry, boysenberry	Herbaceous
2-Methyl-1-butanol	C_5_H_12_O	88.15	1.290	4.760	Bilberry, plum	Alcoholic, ripe fruit, burnt
3-Methyl-1-butanol	C_5_H_12_O	88.15	1.160	2.370	Apple, banana, blackberry,	Whiskey, malt
2-Pentanol	C_5_H_12_O	88.15	1.190	8.047	Apple, banana, black currant, grape, papaya	Green, fuel oil
*cis*-3-Hexen-1-ol	C_6_H_12_O	100.16	1.697	1.039	Apple	Green, leafy
*trans*-2-Hexen-1-ol	C_6_H_12_O	100.16	1.655	0.873	Black currant, apple, grapefruit, kiwi	Green, leafy
*cis*-2-Hexen-1-ol	C_6_H_12_O	100.16	1.755	0.873	Sour cherry, black currant, blueberry, apple, kiwi, papaya, quince	Green
1-Hexanol	C_6_H_16_O	102.18	2.030	0.947	Bilberry, apple, black currant, grapefruit, guava, orange, papaya, plum	Sweet, alcoholic, fresh-cut grass
Benzyl alcohol	C_7_H_8_O	108.14	1.100	0.094	Apricot, apple, bilberry, blueberry, cranberry, fig, mandarin, papaya, plum,	Floral, sweet, cherry
1-Heptanol	C_7_H_16_O	116.20	2.367	0.325	Strawberry, plum, apricot	Fatty, green, pungent
2-Heptanol	C_7_H_16_O	116.20	2.310	0.886	Clove fruit, banana, coconut	Fruity, herbaceous, musty
Phenylethyl alcohol	C_8_H_10_O	122.17	1.360	0.087	Black walnut, strawberry, red raspberry, plum, pear, orange, lemon, blueberry, banana, apricot	Floral, rose-like, honey
6-Methyl-5-hepten-2-ol	C_8_H_16_O	128.21	2.570	0.362	Citrus fruits	Green
1-Octen-3-ol	C_8_H_16_O	128.21	2.519	0.531	Banana, black currant, strawberry,	Mushroom
1-Octanol	C_8_H_18_O	130.23	3.0	0.079	Grapefruit, guava, lime, mandarin, orange, plum	Sweet, rose-like
Cinnamyl alcohol	C_9_H_10_O	134.18	1.950	0.012	Fig, red raspberry	Floral
1-Nonanol	C_9_H_20_O	144.26	3.770	0.041	Cantaloupe, grapefruit, lime, mandarin, orange, plum, watermelon	Floral
2-Nonanol	C_9_H_20_O	144.26	3.230	0.108	Apple, citrus fruits, strawberry, banana	Fruity, green
4-Phenyl-2-butanol	C_10_H_14_O	150.22	2.131	0.015	Blackberry	Floral
1-Decanol	C_10_H_22_O	158.28	4.570	0.009	Apple, lime, mandarin, pear	Fruity, floral, fatty
2-Undecanol	C_11_H_24_O	172.31	4.249	0.015	Banana, apple, papaya, strawberry	Fruity
Aldehydes						
Acetaldehyde	C_2_H_4_O	44.05	−0.340	902.000	Apple, date palm, fig, guava, mandarin, olive, plum, red raspberry, strawberry	Pungent, overripe, apple
2-Methylbutanal	C_5_H_10_O	86.13	1.267	49.317	Cayenne, grapefruit, apple, papaya, plum	Green, malty
3-Methylbutanal	C_5_H_10_O	86.13	1.267	49.317	Plum, banana, apple	Fresh grass, cocoa
Furfural	C_5_H_4_O_2_	96.09	0.410	2.234	Red raspberry, plum, orange, lime, guava, grapefruit, cranberry, apricot, apple	Almond, bread
2-Hexenal	C_6_H_10_O	98.14	1.790	4.624	Avocado, banana, apricot, apple, bilberry, blueberry, currant, peach, plum	Green, leaf
Hexanal	C_6_H_12_O	100.16	1.780	10.888	Cranberry, guava, orange, papaya, apple, banana, plum, watermelon	Green, unripe fruit, grassy
Benzaldehyde	C_7_H_6_O	106.12	1.480	1.270	Bitter almond, apple, sour cherry, fig, guava, lemon, lime, mandarin, orange, peach, plum, red raspberry	Almond, burnt sugar
5-Methyl-2-furfural	C_6_H_6_O_2_	110.11	0.670	0.644	Blackberry, cloudberry, cranberry, mango, red raspberry	Almond, caramel
*trans*, *trans*-2,4-Heptadienal	C_7_H_10_O	110.16	1.891	1.044	Cranberry, bilberry, plum	Fatty, green
Heptanal	C_7_H_14_O	114.19	2.442	3.854	Orange, strawberry, plum, papaya, guava	Fatty, pungent
5-(Hydroxymethyl)furfural	C_6_H_6_O_3_	126.11	−0.778	0.001	Pineapple, cloudberry, roasted almond	Floral
Octanal	C_8_H_16_O	128.21	2.951	2.068	Guava, lime, mandarin, orange, papaya, plum, tangerine, lemon	Lemon, soap
*trans*-2-Nonenal	C_6_H_16_O	140.23	3.319	0.256	Grapefruit, peach, strawberry	Fatty
Ketones						
2-Butanone	C_4_H_8_O	72.11	0.290	90.600	Black currant, plum, apple, guava	Floral, vegetable
2-Pentanone	C_5_H_10_O	86.13	0.910	38.577	Banana, apple, pineapple	Fruity
Acetoin	C_4_H_8_O_2_	88.11	−0.360	2.690	Currant, fig, apple, plum, red raspberry	Buttery
2-Heptanone	C_7_H_14_O	114.19	1.980	4.732	Sour cherry, papaya, pear, berries	Fruity
Furaneol	C_6_H_8_O_3_	128.13	−0.076	0.032	Strawberry, roasted almond, guava, grapefruit, mango, pineapple, red raspberry	Strawberry, sweet, caramel
2-Octanone	C_8_H_16_O	128.21	2.370	1.725	Clove fruit, banana	Soap, gasoline, cheesy, fatty, green
2-Nonanone	C_9_H_18_O	142.24	3.140	0.645	Clove fruit, strawberry	Fruity, green
2-Undecanone	C_11_H_22_O	170.30	4.090	0.098	Banana, guava, strawberry	Fruity
β-Damascenone	C_13_H_18_O	190.29	4.042	0.020	Strawberry, red raspberry, black currant, apricot	Sweet, fruity, apple
α-Ionone	C_13_H_20_O	192.30	3.995	0.014	Blackberry, roasted almond, black currant, plum, red raspberry	Berry, woody
β-Ionone	C_13_H_20_O	192.30	3.995	0.017	Roasted almond, grape, guava, papaya, peach, red raspberry, watermelon	Fruity, woody
Terpenes and terpenoids						
α-Pinene	C_10_H_16_	136.24	4.830	4.750	Apple, blackberry, blueberry, guava, lemon, lime, mandarin, orange, plum,	Woody, resinous
β-Pinene	C_10_H_16_	136.24	4.160	2.930	Black currant, guava, lime, mandarin, orange, plum	Woody, resinous
Camphene	C_10_H_16_	136.24	4.350	3.000	Black currant, lime, mandarin, orange	Woody
α-Phellandrene	C_10_H_16_	136.24	4.408	1.856	Lime, mandarin, orange, papaya	Sweet
Limonene	C_10_H_16_	136.24	4.570	1.550	Blueberry, coconut, guava, lemon, lime, mandarin, orange, plum	Citrus, minty
Sabinene	C_10_H_16_	136.24	3.940	2.633	Red raspberry, orange, mandarin, lime, lemon, grapefruit	Woody
γ-Terpinene	C_10_H_16_	136.24	4.500	1.075	Grapefruit, lemon, lime, mandarin, orange, papaya	Fruity, lemon-like
p-Cymene	C_10_H_14_	134.22	4.100	1.460	Apricot, blackberry, black currant, guava, mandarin, pineapple	Carrot-like
α-Terpinolene	C_10_H_16_	136.24	4.470	1.126	Apricot, blueberry, cherry, coconut, black currant, lemon, lime, mandarin, orange, peach, pineapple, red raspberry	Sweet, piney
Myrcene	C_10_H_16_	136.24	4.170	2.290	Blueberry, black currant, guava, lime, mandarin, orange, papaya	Balsamic
p-Cymen-8-ol	C_10_H_14_O	150.22	2.251	0.020	Bilberry, currant	Musty
Carvone	C_10_H_14_O	150.22	3.070	0.160	Mandarin, orange, plum	Minty
Myrtenal	C_10_H_14_O	150.22	2.980	0.145	Black pepper fruit	Spicy, cinnamon
Myrtenol	C_10_H_16_O	152.24	3.220	0.018	Bilberry, cranberry, red raspberry, wild strawberry	Flowery, minty, medicinal, woody
Camphor	C_10_H_16_O	152.24	2.380	0.650	Coriander fruit	Medicinal, woody
1-Terpineol	C_10_H_18_O	154.25	2.538	0.032	Apple, blueberry, cherry, coconut, cranberry, black currant, guava, lime, mandarin, orange, papaya, peach, pineapple, plum	Woody, musty
*cis*-Rose oxide	C_10_H_18_O	154.25	3.126	0.551	Lychee	Floral, green
4-Terpineol	C_10_H_18_O	154.25	3.260	0.048	Apple, sour cherry, black currant, lime, mandarin, orange, papaya, pineapple, plum	Spicy
Linalool	C_10_H_18_O	154.25	2.970	0.016	Apricot, blueberry, sour cherry, cranberry, fig, grape, lime, nectarine, orange, papaya, pineapple, plum	Floral, rosy, green, fruity, citrus
α-Terpineol	C_10_H_18_O	154.25	2.670	0.028	Apple, apricot, blueberry, cherry, coconut, black currant, guava, lemon, lime, mandarin, orange, papaya, peach, pineapple, plum, red raspberry	Floral, green, fruity
Nerol	C_10_H_18_O	154.25	3.470	0.013	Blueberry, currant, lemon, lime, mandarin, orange, plum	Orange flowers, rose
Geraniol	C_10_H_18_O	154.25	3.560	0.021	Apricot, blackberry, blueberry, boysenberry, grape, lemon, mandarin, orange, plum, red raspberry	Roses, geranium
Citronellol	C_10_H_20_O	156.27	3.300	0.020	Apricot, apple, blueberry, lychee, mandarin, mango, orange	Sweet, floral, rose, citrus
Linalool oxide	C_10_H_18_O_2_	170.25	1.375	0.002	Currant, grape, apple, apricot, blackberry, cloudberry, lychee, papaya, pineapple, red raspberry	Woody, floral
Theaspirane	C_13_H_22_O	194.32	4.204	0.028	Blackberry, grape, guava	Fruity
*trans*, *trans*-α-Farnesene	C_15_H_24_	204.36	6.139	0.010	Apple, grape, guava, lime, mandarin, orange, pear	Sweet, flowery
Esters						
Ethyl acetate	C_4_H_8_O_2_	88.11	0.730	111.716	Pineapple, apple, fig, guava, black currant, papaya, peach, red raspberry	Fruity, floral, pineapple
Ethyl butyrate	C_6_H_12_O_2_	116.16	1.804	12.800	Grapefruit, guava, fig, kiwi, mango, papaya, pineapple, plum, wild strawberry, banana, apple	Fruity, sweet, pineapple, apple
Butyl acetate	C_6_H_12_O_2_	116.16	1.780	11.500	Apple, banana, cherry, black currant, grape, guava, pear, plum, peach, red raspberry, wild strawberry	Fruity, apple, pineapple
Ethyl lactate	C_5_H_10_O_3_	118.13	−0.039	1.163	Apple, grape, apricot, pineapple, plum, red raspberry	Green, fruity
Isoamyl acetate	C_7_H_14_O_2_	130.19	2.260	5.600	Banana, apple, fig, guava, papaya, plum, wild strawberry	Fruity
Ethyl 2-methylbutanoate	C_7_H_14_O_2_	130.19	2.158	7.853	Bilberry, apple, fig, orange, pineapple, plum	Fruity, pineapple
Ethyl 3-methylbutanoate	C_7_H_14_O_2_	130.19	2.158	7.853	Bilberry, plum, pineapple, wild strawberry, apple	Berry
Ethyl 3-hydroxybutanoate	C_6_H_12_O_3_	132.16	0.098	0.362	Grape, blackberry, guava, mango	Fruity, green
Methyl benzoate	C_8_H_8_O_2_	136.15	2.120	0.380	Bilberry, apple, black currant, kiwi	Flowery, honey
*trans*-2-Hexenyl acetate	C_8_H_14_O_2_	142.20	2.580	1.868	Cranberry, guava, mango, apple, banana, peach, pear, plum, wild strawberry	Fruity, green
Hexyl acetate	C_8_H_16_O_2_	144.21	2.870	1.391	Apple, banana, guava, mango, papaya, peach, pear, plum, wild strawberry	Fruity, apple, cherry, pear
Ethyl hexanoate	C_8_H_16_O_2_	144.21	2.823	1.665	Kiwi, guava, apple, banana, black currant, pineapple, plum, wild strawberry	Fruity, pineapple, banana, apple
Ethyl benzoate	C_9_H_10_O_2_	150.18	2.640	0.267	Apple, banana, sweet cherry, cranberry, black currant, grape, kiwi, papaya, peach, red raspberry	Flowery, honey
Methyl salicylate	C_8_H_8_O_3_	152.15	2.550	0.034	Bilberry, black currant, red currant, mandarin, orange, papaya, peach, plum, red raspberry	Green, peppermint
Ethyl heptanoate	C_9_H_18_O_2_	158.24	3.333	0.680	Plum	Fruity, apple
Methyl octanoate	C_9_H_18_O_2_	158.24	3.333	0.523	Wild strawberry, plum, pineapple, papaya	Green, waxy
Phenethyl acetate	C_10_H_12_O_2_	164.20	2.300	0.056	Grape, apple, guava, plum, pineapple	Fruity, rose, floral, honey
Ethyl decanoate	C_12_H_24_O_2_	200.32	4.861	0.034	Banana, cherry, citrus fruits, grape, guava, pear, pineapple, plum, plumcot, wild strawberry	Fruity, grape
Ethyl octanoate	C_10_H_20_O_2_	172.27	3.842	0.224	Plum, plumcot, guava	Fruity, sweet, banana, pineapple
Diethyl succinate	C_8_H_14_O_4_	174.20	1.260	0.439	Grape, apple, cocoa	Wine, overripe merlon, lavander, fruity
Ethyl cinnamate	C_11_H_12_O_2_	176.22	2.990	0.003	Currant, guava, peach, wild strawberry	Fruity, honey, cinnamon
Ethyl 3-phenylpropanoate	C_11_H_14_O_2_	178.23	2.730	0.028	Muskmelon	Floral
Ethyl laurate	C_14_H_28_O_2_	228.38	5.710	0.007	Grape, guava, pear, wild strawberry	Waxy, fruity, floral, leaf
Ethyl hexadecanoate	C_18_H_36_O_2_	284.48	7.918	0.000	Apricot, guava,	Waxy
Phenols						
Phenol	C_6_H_6_O	94.11	1.460	0.614	Blueberry, cranberry, guava	Medicinal
4-Ethylphenol	C_8_H_10_O	122.17	2.580	0.083	Cranberry	Smoky
4-Methylguaiacol	C_8_H_10_O_2_	138.17	1.925	0.078	Cocoa	Spicy, smoky
4-Vinylguaiacol	C_9_H_10_O_2_	150.18	2.573	0.019	Apple, wild strawberry	Woody, smoky
Vanillin	C_8_H_8_O_3_	152.15	1.210	0.002	Blueberry, clove fruit, grape, pineapple, wild strawberry	Sweet, creamy, vanilla
Eugenol	C_10_H_12_O_2_	164.20	2.270	0.010	Blueberry, clove fruit, cranberry, black currant, fig, guava, peach, plum, red raspberry	Clove, spicy, pungent
Methyl eugenol	C_11_H_14_O_2_	178.23	2.973	0.027	Apricot, banana, clove fruit, nutmeg, passion, papaya, peach, red raspberry	Spicy, clove
Elemicin	C_12_H_16_O_3_	208.26	2.298	0.007	Nutmeg	Woody, floral
Lactones						
γ-Butyrolactone	C_4_H_6_O_2_	86.09	−0.640	0.450	Mango, pineapple	Creamy, caramel, sweet
γ-Nonalactone	C_9_H_16_O_2_	156.22	1.942	0.009	Apricot, coconut, black currant, papaya, pineapple, plum	Coconut

VP–vapor pressure at 25 °C (mmHg); fruit source for flavor compounds as well as VP, logP, MW and formula were obtained from http://www.thegoodscentscompany.com and https://pubchem.ncbi.nlm.nih.gov (accessed on 1 July 2021).

**Table 2:** Median lethal dose (LD_50_) of flavor compounds (FC).

FC	LD_50_ (mg/kg)	FC	LD_50_ (mg/kg)	FC	LD_50_ (mg/kg)
Acetic acid	3310 ^1,a^	2-Hexenal	290 ^2,b^, 780 ^1,a^	*cis*-Rose oxide	4300 ^1,a^
Butanoic acid	3180 ^2,b^	Hexanal	4890 ^1,a^, 8292 ^2,a^	4-Terpineol	1300 ^1,a^, 1016^2,a^
2-Methylbutanoic acid	4100 ^1,a^	Benzaldehyde	1300 ^1,a^, 28 ^2,a^	Linalool	2200 ^2,a^, 8000 ^2,b^
3-Methylbutanoic acid	1120 ^2,c^	5-Methyl-2-furfural	2200 ^1,a^	α-Terpineol	4300 ^1,a^
Hexanoic acid	3180 ^2,b^, 1725 ^2,c^	*trans*, *trans*-2,4-Heptadienal	1150 ^1,a^	Nerol	4500 ^1,a^
Octanoic acid	10080 ^1,a^, 600 ^2,c^	Heptanal	3200 ^1,a^	Geraniol	3600 ^1,a^
Decanoic acid	500 ^1,b^, 129 ^2,c^	5-(Hydroxymethyl)furfural	2500 ^1,a^	Citronellol	3450 ^1,a^
Ethanol	7060 ^1,a^, 1440 ^1,c^	Octanal	5630 ^1,a^	Linalool oxide	1150 ^1,a^
1-Propanol	1870 ^1,a^, 483 ^3,c^	*trans*-2-Nonenal	3700 ^3,d^	Ethyl acetate	5620 ^1,a^, 4935 ^3,a^
2-Butanol	2193 ^1,a^	2-Butanone	2737 ^1,a^, 4050 ^2,a^	Ethyl butyrate	13,000 ^1,a^, 5228 ^3,a^
2-Methyl-1-propanol	2460 ^1,a^, 340 ^1,c^	2-Pentanone	1600 ^1,a^, 1600 ^2,a^	Butyl acetate	10,768 ^1,a^, 3200 ^3,a^
1-Butanol	790 ^1,a^, 310 ^1,c^	Acetoin	>5000 ^1,a^	Ethyl lactate	8200 ^1,a^, 2500 ^2,a^
2-Methyl-3-buten-2-ol	1315 ^1,b^, 800 ^2,b^	2-Heptanone	1670 ^1,a^	Isoamyl acetate	16,600 ^1,a^, 7422 ^3,a^
3-Methyl-3-buten-1-ol	3652 ^1,a^	Furaneol	1608 ^2,a^	Ethyl 3-methylbutanoate	>5000 ^1,a^
3-Methyl-1-butanol	1300 ^1,a^	2-Octanone	3089 ^1,a^, 800 ^1,b^	Methyl benzoate	2170 ^1,a^, 2170 ^3,a^
2-Pentanol	2821 ^3,a^	2-Nonanone	3200 ^1,a^, 7994 ^2,a^	*trans*-2-Hexenyl acetate	> 5000 ^1,a^
*cis*-3-Hexen-1-ol	4700 ^1,a^	2-Undecanone	3880 ^2,a^	Hexyl acetate	> 5000 ^3,a^
*trans*-2-Hexen-1-ol	3500 ^1,a^	α-Ionone	2277 ^2,b^	Ethyl benzoate	2100 ^1,a^
1-Hexanol	720 ^1,a^	β-Ionone	4590 ^1,a^	Methyl salicylate	1220 ^1,a^
Benzyl alcohol	1230 ^1,a^	α-Pinene	3700 ^1,a^	Ethyl heptanoate	>34,640 ^1,a^
1-Heptanol	500 ^1,a^, 1500 ^2,a^	β-Pinene	4700 ^1,a^	Phenethyl acetate	5200 ^1,a^
2-Heptanol	2580 ^1,a^	Camphene	>5000 ^1,a^	Ethyl octanoate	25,960 ^1,a^
Phenylethyl alcohol	1790 ^1,a^, 2540 ^2,a^	α-Phellandrene	5700 ^1,a^	Diethyl succinate	8530 ^1,a^
1-Octen-3-ol	340 ^1,a^, 56 ^2,c^	Limonene	5300 ^1,a^	Ethyl cinnamate	4000 ^2,a^, 4000 ^1,a^
1-Octanol	1790 ^2,a^, 69 ^2,c^	γ-Terpinene	3650 ^1,a^	Phenol	317 ^1,a^, 270 ^2,a^, 127 ^1,b^
Cinnamyl alcohol	2675 ^2,a^	p-Cymene	4750 ^1,a^	4-Ethylphenol	>5000 ^1,a^, 138 ^2,b^
1-Nonanol	3560 ^1,a^, 6400 ^2,a^	α-Terpinolene	5170 ^1,a^, 2830 ^2,a^	4-Methylguaiacol	740 ^1,a^, 76 ^2,c^
1-Decanol	4720 ^1,a^, 6500 ^3^	Myrcene	>5000 ^1,a^	Vanillin	475^2,b^, 4370^1,a^
Acetaldehyde	661 ^1,a^	Carvone	1640 ^1,a^	Eugenol	1930 ^1,a^, 500 ^2,b^, 72 ^2,c^
2-Methylbutanal	6400 ^1,a^	Myrtenal	2300 ^1,a^, 170 ^2,c^	Methyl eugenol	810 ^1,a^, 540 ^2,b^, 112 ^2,c^
3-Methylbutanal	5600 ^1,a^, 4750 ^2,a^	Camphor	1310 ^2,a^, 3000 ^2,b^	γ-Butyrolactone	1540 ^1,a^, 1000 ^1,b^
Furfural	65 ^1,a^,20 ^1,b^	1-Terpineol	4300 ^1,a^	γ-Nonalactone	6600 ^1,a^

Letters and numbers in superscript have the following meanings: ^1^—rat, ^2^—mouse, ^3^—rabbit, ^a^—oral, ^b^—intraperitoneal, ^c^–intravenous; data were obtained from http://www.thegoodscentscompany.com and https://pubchem.ncbi.nlm.nih.gov (accessed on 1 July 2021).

**Table 3:** Flavor compounds complexed with starch.

Flavor Compounds	Type of Starch	Applied Technique	Applied Methods of Analysis	Reference
D-limonene, ethyl hexanoate, octanal and 1-hexanol	Seven different starch materials	Extrusion	Inverse gas chromatography	[51]
1-Hexanol, 2-hexanol, D-limonene, ethyl hexanoate and octanal	Native corn starch	Not specified	Inverse gas chromatography	[50]
Vanillin	Oxidized starch from corn and waxy amaranth starch	Spray-drying	Spectrophotometric analysis	[52]
Hexanal and menthone	Non-modified and modified tapioca starch	Not specified	Proton transfer reaction mass spectrometry	[46]
Methyl phenylacetate, 3-hexanol, ethyl 2-methylbutanoate, ethyl pentanoate, methyl hexanoate, ethyl hexanoate, hexyl acetate, isopropyl propionate, ethyl butanoate and ethyl 3-methylbutanoate	Different commercially available starches	Not specified	Gas chromatography-mass spectrometry analysis with solid-phase micro-extraction	[43]
Menthone	Starch	Freeze-drying	Transmission electron microscopy, dynamic light scattering, X-ray diffraction, differential scanning calorimetry, Fourier transform infrared spectroscopy, high-performance size exclusion chromatography	[53]
Menthol	Modified starch, gelatin, oil–gelatin emulsion and aquacoat	Spray-drying	Headspace gas chromatography, encapsulation efficiency, dynamic viscosity, density, tension	[54]
Ethyl acetate, R-(+)-limonene and hexanal	Waxy maize starch and potato starch	Not specified	Headspace gas chromatography-mass spectrometry	[55]
1-Decanol, 1-decanal, 1-naphthol, decanoic acid, δ-decalactone, L-menthone, L-menthol, Γ-decalactone and thymol	High-amylose maize starch	Freeze-drying	X-ray diffraction, differential scanning calorimetry, Fourier transform infrared spectroscopy, solid-state ^13^C nuclear magnetic resonance spectroscopy and molecular dynamics simulation	[10]
1-Hexanol, hexanal, *trans*-2-hexanal and 2-hexanone	Potato starch and corn starch	Not specified	Differential scanning calorimetry and X-ray diffraction	[44]
Limonene, menthol and menthone	Waxy starch, corn starch, high amylose corn starch and amylose (type III from potato)	Freeze-drying	X-ray diffraction, differential scanning calorimetry, dynamic light scattering and atomic force microscopy	[56]
Menthol	High amylose maize starch (six different V-type crystalline structures)	Molecular inclusion	X-ray diffraction, gas chromatography-mass spectrometry and differential scanning calorimetry	[57]
Ethyl butyrate, ethyl hexanoate, methyl cinnamate, (*Z*)-hex-3-en-1-ol, linalool, vanillin and δ -decalactone	Cross-linked waxy corn starch, carrageenan and sucrose	Not specified	Gas chromatography	[58]
Diacetyl, 2-butanone, hexanal, 2-pentanol, ethyl acetate, ethyl butyrate, 1-hexanol, heptanal, 2-heptanone, 2-octanone, octanal, dimethyl sulphide, α-pinene, propyl acetate, 1-propanol, 1-butanol, 3-methyl-1-butanol, butyl acetate, 2-nonanol and 2-decanone	Potato starch and potato amylopectin	Not specified	Gas chromatography-mass spectrometry	[47]
Heptanolide, menthone, linalool, menthol, heptanol and carvone	Corn starch	Freeze-drying	Scanning electron microscopy, differential scanning calorimetry, X-ray diffractometry, Fourier transform infrared spectroscopy, nuclear magnetic resonance spectroscopy	[59]
Isoamyl acetate, ethyl hexanoate and linalool	Four corn starches and potato amylose	Not specified	Headspace gas chromatography, X-ray diffractometry, differential scanning calorimetry, rheology measurement	[17]

**Table 4:** Flavor compounds complexed with maltodextrin.

Flavor Compounds	Used Material	Applied Technique	Applied Methods of Analysis	Reference
1-Propanol, diacetyl, 2-pentanone, hexanal and 2-heptanone	Maltodextrin	Freeze–thawing	Differential scanning calorimetry, gas chromatography headspace analysis, creaming stability, Microstructural observation, oiling off, emulsion flavoring and particle size analysis	[63]
Asparagus juice flavor	Maltodextrin	Spray-drying	Gas chromatography-mass spectrometry, moisture content, glass transition temperature, particle size distribution, morphology	[64]
Citral	Maltodextrin, sucrose and trehalose	Spray-drying	Droplet size, viscosity, molecular mobility, microstructure	[35]
Picrocrocin, safranal and crocin	Maltodextrin, pectin and whey protein concentrate	Multiple emulsification	Emulsion droplet size analysis, stability measurement, encapsulation efficiency and release characteristics	[65]
Picrocrocin, safranal and crocin	Maltodextrin, gum arabic and gelatine	Spray-drying	Encapsulation efficiency, powder yield determination, moisture content, scanning electron microscopy	[66]
Orange terpenes	Maltodextrin and sucrose	Hot melt counter-rotating extrusion	Differential scanning calorimetry, X-ray diffractometry, gas chromatography, incident light and polarization microscopy	[67]
Orange terpenes and carvacrol	Maltodextrin and sucrose	Batch mixing	Water content, differential scanning calorimetry, X-ray diffractometry and gas chromatography	[68]
Isoamyl acetate, allyl caproate, linalool, orange oil and citral	Gum arabic, maltodextrin and sodium caseinate	Spray-drying	Gas chromatography-mass spectrometry, scanning electron microscopy, physical properties (encapsulation efficiency, viscosity, moisture, emulsion stability), nonenzymatic browning	[69]
1,8-Cineole, camphor and α-pinene	Maltodextrin, gum arabic, modified starch, inulin	Spray-drying	Gas chromatography-mass spectrometry, differential scanning calorimetry, scanning electron microscopy, characterization of the microcapsules (wettability and solubility, moisture content, bulk density, oil retention)	[60]
Cocoa flavor	Maltodextrin and Hi-Cap 100	Spray-drying	Gas chromatography-olfactometry, gas chromatography-mass spectrometry, Fourier transform infrared spectroscopy, process yield, moisture and water activity, tapped density, hygroscopicity, water solubility index and water absorption index, color parameters, morphology and size particle, sensory evaluation	[39]
Orange oil flavor compounds	Maltodextrin, sucrose, trehalose, lactose, modified starch and gum arabic	Spray-drying	Electronic nose and sensory analysis	[70]

**Table 5:** Flavor compounds complexed with cyclodextrin.

Flavor Compounds	Type of Cyclodextrin	Applied Technique	Applied Method of Analysis	Reference
Menthol, D-limonene, (+)-limonene, (-)-limonene, hydroxycitronellal, (+/-)-linalyl acetate, α-ionone, vanillin and γ-decalactone	β-cyclodextrin	Crystallization from the ethanol–water solution	Gas chromatography and thermal gravimetric analysis	[85]
Thymol and cinnamaldehyde	β-cyclodextrin	Freeze-drying	Differential scanning calorimetry, release studies, moisture sorption properties	[86]
Thymol and thyme essential oil	β-cyclodextrin	Freeze-drying and kneading	Entrapment efficiency, differential scanning calorimetry, phase solubility, particle size and morphology	[87]
α-Terpineol	β-cyclodextrin, 2-hydroxypropyl-β-cyclodextrin	Freeze-drying	Differential scanning calorimetry, scanning electron microscopy, water sorption isotherms and water content analysis, storage study	[88,89]
Linalool	2-hydroxypropyl-β-cyclodextrin	Freeze-drying	High-performance liquid chromatography, ^1^H NMR spectroscopy, circular dichroism spectroscopy, solubility, stability and release profiles studies	[90]
(+)-Linalool and (-)-linalool	α- and β-cyclodextrin	Crystallization from the ethanol–water solution	Gas chromatography and thermal gravimetric analysis	[85]
(+)-Isopulegole and (-)-isopulegole	β-cyclodextrin	Molecular inclusion	High-performance liquid chromatography, X-ray crystallography	[91]
Eugenol	α-, β-, γ- and 2-hydroxypropyl-β-cyclodextrin	Freeze-drying	Fourier transform infrared spectroscopy, differential scanning calorimetry, thermal gravimetric analysis	[92]
Eugenol	β-cyclodextrin	Freeze-drying	Oxidative differential scanning calorimetry, particle size analysis and morphology, entrapment efficiency, phase solubility studies	[93]
Ethyl benzoate	2-hydroxypropyl- β-cyclodextrin	Freeze-drying	UV/Vis spectroscopy, Fourier transform infrared spectroscopy, phase solubility, molecular modeling and controlled release studies	[94]
Estragole	α-, β-, γ-, 2-hydroxypropyl-β-, low methylated-β and randomly methylated-β-cyclodextrin	Freeze-drying	Static headspace gas chromatography, UV/Vis spectroscopy, ^1^H NMR spectroscopy, encapsulation efficiency, differential scanning calorimetry, Fourier transform infrared spectroscopy	[95]
Citronellal and citronellol	β-cyclodextrin	Kneading	Gas chromatography-mass spectrometry, scanning electron microscopy, Fourier transform infrared spectroscopy, differential scanning calorimetry	[96]
L-Menthol, ethyl butyrate, ethyl hexanoate, citral, benzaldehyde and methyl anthranilate	α-, β- and γ-cyclodextrin	Molecular inclusion	Headspace gas chromatography and sensory evaluation	[97]
Turmeric extract (Curcuminoids)	β-cyclodextrin and brown rice flour	Spray-drying	Gas chromatography-mass spectrometry, high-performance liquid chromatography, product recovery, moisture content, hygroscopicity, encapsulation efficiency, scanning electron microscopy, sensory analysis	[38]
Geraniol	γ –cyclodextrin and polyvinyl alcohol	Electro-spinning	X-ray diffraction, thermal gravimetric analysis, ^1^H NMR spectroscopy, scanning electron microscopy	[98]
Sweet orange flavor (sweet orange oil, ethyl maltol, decanal, linalool, lemon oil, carvone, ethyl butyrate and benzyl alcohol)	β-cyclodextrin	Molecular inclusion	Thermal gravimetric analysis and optical microscopy analysis	[99]

## Data Availability

Not applicable.
